# Effectiveness of a Community Pharmacy-Based Health Promotion Program on Hypertension in Bangladesh and Pakistan: Study Protocol for a Cluster-Randomized Controlled Trial

**DOI:** 10.3390/healthcare12141402

**Published:** 2024-07-15

**Authors:** Md. Mizanur Rahman, Ryota Nakamura, Md. Monirul Islam, Md. Ashraful Alam, Syed Khurram Azmat, Motohiro Sato

**Affiliations:** 1 Research Center for Health Policy and Economics, Hitotsubashi University, Tokyo 186-8601, Japan; 2 Graduate School of Economics, Hitotsubashi University, Tokyo 186-8601, Japan; 3 Global Public Health Research Foundation, Dhaka 1230, Bangladesh; 4 Centre for Policy Studies, University College Cork, T12 K8AF Cork, Ireland; 5 Department of Computational Diagnostic Radiology and Preventive Medicine, The University of Tokyo Hospital, Tokyo 113-8655, Japan; 6 AAPNA-Institute of Public Health, Jinnah Sindh Medical University, Karachi 75510, Pakistan

**Keywords:** hypertension control, community pharmacy, cost-effectiveness, South Asia, protocol

## Abstract

The aim of this multi-country, cluster-randomized trial is to test the impact of pharmacy-based health promotion to reduce the blood pressure of individuals with hypertension over a 12-month period in Bangladesh and Pakistan. The trial will be implemented with two arms. In Bangladesh, the estimated sample size is around 3600 hypertensive patients. In Pakistan, we will select samples equivalent to 10% of the participants from Bangladesh, comprising 360 hypertensive patients from four pharmacies. Community pharmacies will be randomized into one of two parallel groups (allocation ratio 1:1). Pharmacy professionals in the treatment arm will provide their patients with educational training and counseling, as well as phone calls/mobile text messages and care coordination in the health sector, as part of the intervention. The study will be conducted in three phases: a baseline survey with intervention, a midline survey with intervention and follow-up, and an endline survey with impact evaluation. The primary outcome of the study will be BP. The secondary outcomes will be BP controlled to target, treatment adherence, quality of life, mortality or hospital admission rates resulting from hypertension and its related complications, incremental cost per health-related quality of life gained, knowledge on healthy lifestyle and dietary behavior, and change in the prevalence of current smoking status.

## 1. Introduction

Globally, hypertension is a leading cause of cardiovascular disease and death [1]. Approximately two-thirds of the world’s 1.28 billion hypertensive patients live in low- and middle-income countries (LMICs) [2]. South Asian countries have experienced a steep rise in hypertension prevalence, with Bangladesh, India, and Pakistan having 48.0%, 52.3%, and 46.2% [3,4,5]. Hypertension in low-income countries is disproportionately caused by rapid urbanization, unhealthy lifestyles, and inactivity [6,7]. In LMICs, awareness, treatment, and control of hypertension remain unacceptably low, mostly because of poor medication adherence, limited health resources, and difficulties accessing healthcare [8,9]. Furthermore, a systematic review suggested that factors such as inadequate healthcare information, dissatisfaction with the services provided, and fewer interactions with physicians contribute significantly to medication nonadherence [10]. LMICs with weak health literacy, poor health systems, and high cardiovascular disease fatality rates have a disproportionately high prevalence of hypertension [11,12]. Consequently, most hypertensive patients take medications only when symptomatic and lead sedentary lifestyles despite being aware of dietary recommendations [13,14].

The American Heart Association (AHA) and World Health Organization (WHO) have set specific blood pressure targets to reduce the risk of cardiovascular events [2,15]. The AHA defines Stage 2 hypertension as having a systolic blood pressure (SBP) of 140 mmHg or higher or a diastolic blood pressure (DBP) of 90 mmHg or higher, indicating that these levels are significant thresholds for initiating treatment to prevent complications [15]. Lifestyle and medication adherence can be improved by educating the patients [15,16,17,18,19]. Using the available non-physician health workforce in LMICs is a logical step for hypertension management, where doctors are often scarce. The approach could be cost-effective and essential for resource-constrained countries like Bangladesh, India, and Pakistan. In addition to many non-physician healthcare providers (HCPs), community pharmacists (CPs) are widely recognized as valuable sources of reliable information for people of all ages [19,20]. Pharmacies can be the first contact points for patients seeking primary healthcare services, due to their relatively convenient community location. They also assist hypertensive patients in achieving their treatment goals with individualized advice and education, increasing patients’ knowledge for better management of hypertension [21]. The results of a meta-analysis showed that CPs successfully reduced systolic blood pressure (SBP) and diastolic blood pressure (DBP) by 9.3 mmHg and 3.6 mmHg, respectively, over a one-year period [17].

As of today, only a small number of studies in Pakistan and India have demonstrated the above findings, including improved control of blood pressure, medication adherence, and understanding of hypertension [22,23,24,25,26]. To our knowledge, no study has examined this unique intervention’s cost-effectiveness and impact on mortality and hospital admission rates in Bangladesh and Pakistan. This information is crucial to informing policymakers on the potential of CP as an efficient and high-quality care solution compared to physician-centered care. Due to their similarities and differences in culture, religion, socioeconomic background, and other aspects, the findings of this study will be adaptable, transferable, and scalable to different settings or other LMICs, preventing numerous premature deaths, strokes, and heart attacks every year.

## 2. Aim and Hypothesis

In this study, we will test the effectiveness and cost-effectiveness of enhanced pharmacy-based care provided to community people in Bangladesh and Pakistan on blood pressure control and treatment adherence. This is the first multi-country trial to evaluate pharmacy-based health promotion in these two countries, focusing on the role of CP in hypertension treatment.

### 2.1. Hypothesis

The study hypothesizes that hypertensive patients receiving the community pharmacy-based health intervention will achieve the following outcomes:

- Reduced systolic and/or diastolic BP;
- Improved treatment adherence and BP control to target levels;
- Reduced hospitalization and death due to cardiovascular events;
- Positive changes in lifestyle behaviors;
- Increased in HRQoL.

### 2.2. Objectives

This study evaluates the effectiveness of community pharmacy-based interventions in controlling BP among hypertensive patients in Bangladesh and Pakistan. The study assesses both clinical outcomes and cost-effectiveness. The primary objective is to evaluate the impact of patient counseling and education by CPs on BP control and treatment adherence, followed by a cost-effectiveness analysis to demonstrate the value for money in public spending on hypertensive care. Specifically, the cost-effectiveness analysis will calculate the cost per unit (mmHg) of blood pressure reduction and the cost per HRQoL gained.

## 3. Methods

### 3.1. Study Design and Settings

**Figure 1:**
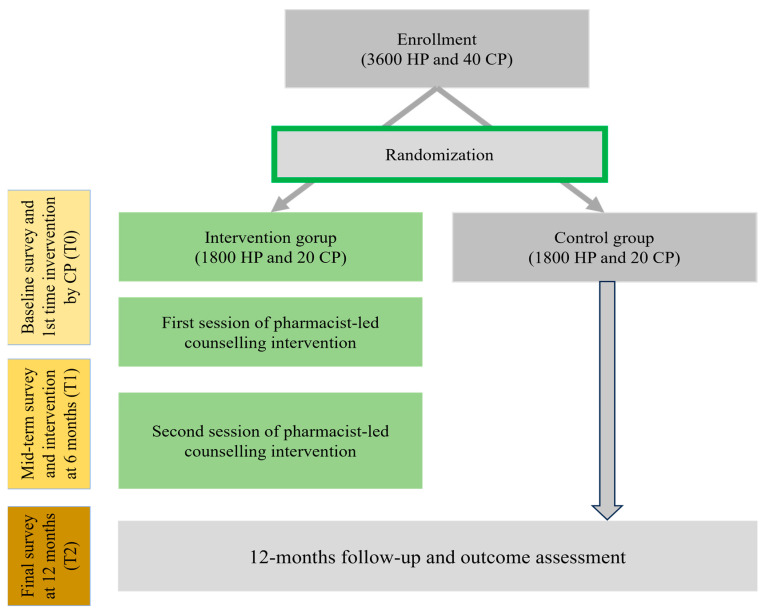
Study design and workflow, Bangladesh. HP, hypertensive patients; CP, community pharmacy.

A two-arm parallel-group cluster-randomized controlled trial (cRCT) will be conducted among hypertensive patients in Bangladesh and Pakistan. In the first stage, two districts in Bangladesh were chosen randomly: Rangpur district and Chuadanga district. In the second stage, from each district, one subdistrict was selected randomly: Pirganj and Alamdanga. Finally, 20 pharmacies were chosen from each subdistrict to reach our target sample. The overall flow of the study is shown in Figure 1. Randomization clusters are community pharmacies. Each country will follow a two-arm cRCT design. According to a 4.65% BP reduction with a standard deviation of 49.78 [27], the estimated sample size for Bangladesh is around 3600 at 80% power and 5% significance. Approximately 10% of participants will be selected based on a Bangladesh sample from Pakistan (360 hypertensive patients from four pharmacies). The average number of hypertensive patients per pharmacy is 90. Due to financial limitations and logistical challenges, it is necessary to choose 10% of the participants from Pakistan. This decision was primarily influenced by the availability of resources and the specific focus of our research project. These limitations included budget constraints that impacted the scope of the study and logistical challenges related to the larger geographic and infrastructural differences between the two countries. Conducting a full-scale study in Pakistan similar to Bangladesh is not feasible with the available resources. However, by including participants from Pakistan, we aim to enhance the diversity within our study population, despite some similarities in sociodemographic profiles and health behaviors between the two countries.

### 3.2. Study Participants

As part of the consensus, participants who come to the pharmacy to purchase hypertensive medication will be listed with their basic information. The following information will be needed: their name, address, gender, duration of hypertension, who will be taking the medication, and at least two contact numbers. In order to confirm whether prospective hypertensive adults are hypertensive, their blood pressure will be measured again. Both male and female participants will be invited to participate. The eligibility criteria include (1) aged 18 years and above; (2) hypertensive (defined as (a) SBP ≥ 140 mmHg and DBP ≥ 90 mmHg and/or taking antihypertensive medications); (3) capable of communicating verbally in local language; (4) permanent resident of the study area; (5) access to mobile phone; and (6) consent to participate. Exclusion criteria are (1) pregnant and lactating women; (2) individuals with advanced medical disease (e.g., cancer, heart failure, chronic obstructive pulmonary disease, end-stage renal disease, advanced neurological disease, etc.); (3) those having cognitive and psychiatric problems; (4) involvement in any other interventional study; and (5) individuals who are unable to give informed consent. Pharmacists’ inclusion criteria include (1) ensuring a sufficient amount of time to implement intervention on hypertensive patients; (2) consent to participate; and (3) being able to operate a smartphone or tablet. Screening for patients’ eligibility will be conducted by the CP during regular visits, and upon deeming them eligible, the CP will inform the principal investigator or research assistant of this study to approach the patients, briefly explain the study, and ask them to sign informed consent.

### 3.3. Randomization

Community pharmacies will then be randomized to one of two parallel groups (allocation ratio 1:1) using a computer-generated random number sequence. Participants and pharmacies will be assigned to intervention and control groups in a 1:1 ratio. To reach the target sample, every pharmacy needs to recruit 90 hypotensive patients. The randomization and study procedure are presented in Figure 1.

### 3.4. Blinding

Only the outcome evaluators will be blinded to group allocation due to the nature of the intervention. To prevent contamination, we will select the CPs of the treated group and control from considerably different locations. CP will deliver the intervention, and research assistants (field enumerators and supervisors) will engage in data collection from the participants. However, pharmacists and research assistants will not be blinded since they are involved in interventional processes.

### 3.5. Training of the Pharmacists

Mandatory communication training will enhance treatment integrity since pharmacist skills and competences will determine the quality of the intervention. Before the study begins, CPs in the intervention arm will receive training involving both theoretical and practical sessions. It will cover the prevalence, risk factors, complications, and diagnosis of hypertension, as well as non-pharmacological ways of preventing it. On the practical side, CPs will learn how to measure blood pressure using a manual sphygmomanometer, interpret the measurements, and educate and counsel hypertensive patients through audio–visual presentations. As part of the intervention strategy, blood pressure control, the identification of hypertension-related complications, health education, medication and lifestyle modification counseling, follow-up, and referral to a medical practitioner or hospital will be provided.

### 3.6. Intervention

This project will consist of three phases: baseline survey with first-time intervention implementation; second intervention at 6 months; and third, endline survey at 12 months. The details of the intervention content are mentioned in Table 1. As part of the intervention within the randomized selected groups, the CP will conduct two 15–30 min sessions throughout the intervention period, once at the baseline or first visit (T0) and once at the sixth month (T1). Patients will attend the two sessions at the pharmacies where they collect their prescription medication. Face-to-face counseling sessions will be held about hypertension and its management, including lifestyle changes and drug compliance. Patients will be able to view the educational material in the pharmacy via a tablet since audio–visual presentations make learning easier. The detailed training material and handbook are presented as Supplemental Files S1 and S2. To maintain adherence to intervention, the intervention group will receive phone call intervention at 3 months and 6 months. The patient will be reminded every time about their subsequent visits via phone calls/short message services (SMS). Patients’ BP will be monitored routinely during each session, and information regarding medication and complications will also be obtained. This intervention will be low in cost, aligned with the current CP workflow, and will not require any significant changes in the existing system. Both interventions will be conducted by the same pharmacist to ensure consistency. Furthermore, CPs in the intervention group will receive a checklist of items and a counseling protocol to prepare them for what they need to do during patient visits. A pilot study was conducted in two pharmacies in Bangladesh before starting the study in order to optimize the intervention steps and materials. Nevertheless, there are no restrictions on how many times the patient may meet the pharmacists outside of these ‘core’ consultations. In this case, the patient will decide with the pharmacist according to needs, and it is suggested that this coincides with the monthly collection of prescriptions.

In the control group, routine pharmacy services and counseling will be provided without interference during all pharmacy visits, as per each country’s pharmacy practice guidelines, and they will serve as a comparator group to determine the impact of the intervention implementation. Assessments will be completed at the same time points as those in the intervention group.

### 3.7. Data Collection

Data will be collected primarily through a face-to-face interview with an open-ended structured questionnaire. The interview team will comprise graduates in social science, public health, demography, and statistics with extensive experience in survey techniques, along with two experienced supervisors who will oversee the survey. They will all undergo four days of mandatory training and two days of practical sessions focused on the questionnaire content, techniques to obtain more information from study subjects, and strategies for acquiring complete and valid data. An operational manual will be provided to both interviewers and supervisors to ensure clarity on the research purpose. The supervisor will be responsible for regularly monitoring the intervention process. The details about the data collection time point are mentioned in Table 2.

At baseline, after obtaining informed consent by the interviewer, data will be collected on the self-reported basic socioeconomic and demographic characteristics of the patients, such as age at the completion of the questionnaire, gender, highest level of education completed, occupation, religion, and marital status. Also, the basic characteristics of the CP (age, gender, and working experience in the community pharmacy in years) will be collected. Information on disease profiles will be obtained by face-to-face direct interview and used to inform the consultation at each stage. The following data will be requested from patients and CP at baseline and 12 months via questionnaire or pre-defined data collection form (as appropriate):

- Clinical factors of the patients [time since diagnosis of hypertension in years; types of concomitant antihypertensive medications; development of any hypertension-related complications such as coronary heart disease (CHD), and heart failure and stroke; in the previous six months: (i) occurrence of any adverse health condition related to hypertension requiring hospitalization; (ii) total number of days in hospital following admission; and (iii) total number of visits to doctors for hypertension and its related complication management;
- Organizational information of the community pharmacies (number of visits from hypertensive patients per month in last six months, number of hypertensive cases referred to hospital due to complications in last six months);
- Quality of life measure (EuroQol EQ-5D-5L) [28].

Additional data will be collected by enumerators to enable the calculation of the QRisk2 score, including the following:

- Blood pressure (lowest of two measurements used);
- Fasting blood glucose level;
- Weight and height;
- Smoking status (options aligned with QRisk2).

For the economic evaluation, all costs related to pharmacy-based intervention will be collected including patient counseling and education time and training, telephone and material costs, cost of care at the hospital (for complicated cases requiring visits to physicians and admission), and other administrative costs.

### 3.8. Outcome Evaluation

The primary effectiveness of the outcome will be evaluated by changes in the SBP and DBP of the patients from baseline to final follow-up at 12 months post-intervention. The secondary outcome measures will include (1) BP controlled to target (SBP < 140 mmHg and DBP < 90 mmHg); (2) composite outcome of death (all-cause) or hospital admission due to CHD, heart failure, or stroke; (3) incremental cost per HRQoL gained from baseline to end of follow-up; (4) improvement in the knowledge of patients on healthy lifestyle; (5) change in dietary salt intake; (6) change in quality of life; (7) change in the prevalence of current smokers; and (8) the treatment adherence rate. Hypertensive patients with poor adherence to their treatment regimens experience uncontrolled blood pressure. Adherence to hypertension treatment is defined as following the treatment regimen agreed to with their physician. According to the previous literature [29,30,31], a reduction of 5 mmHg of SBP is considered clinically relevant as it has been found to be significantly associated with a risk reduction in CHD, stroke, and other serious cardiovascular events. Health service utilization parameters will include the number and reason for visits to physicians and hospital admission, and when further complicated, the death of the patient. The cost-effectiveness measures are incremental cost per mmHg BP reduction or cost per HRQoL gained from baseline to end of follow-up at 12 months. For determining dietary salt intake level, a 24 h urinary sodium measurement will be opted.

### 3.9. Cost-Effectiveness Analysis

The intervention cost will be calculated by considering average times (including both patient contact and non-contact) for the pharmacist at each consultation (baseline, at 6 months, at 12 months, and any additional consultations). Participant attendance rates, recorded by the pharmacist delivering the intervention, will be combined with the estimated cost per consultation, allowing for the total cost of all consultations to be calculated for each participant. Pharmacists will receive proper training to convey the intervention using a tablet and other equipment (e.g., booklet, BP record book) from each participating pharmacy. All of these items will be documented along with their associated costs, including pharmacist time (cost calculated as above), equipment costs, and consumables for blood pressure measurement. Pharmacist training costs will also be measured. To calculate the per-patient total intervention costs, all of the above-mentioned costs will be equally divided among all intervention group participants and then added to the aforementioned per-patient total consultation cost. The cost of each activity will be valued using standard rates. Finally, the cost per unit (mmHg) of blood pressure reduction and/or cost per HRQoL gain will also be measured.

### 3.10. Data Management

All data will be confidentially stored in computers and entered manually in spreadsheets. Findings will be available to authorized individuals for analysis and reporting purposes only. To ensure the privacy and accuracy of the study data, the datasheets will be in a password-protected server, and data entry will be reconfirmed and double-checked for any potential errors during input. The research data will be published in a manner that ensures participants’ anonymity. A unique identifier will be used to coordinate participants’ data across baseline and all follow-up stages. Patients will be coded as numbers, with the code key stored in a password-protected document, ensuring all safety precautions. The patients who will complete all the scheduled follow-ups will be included in the analysis of results. To avoid a withdrawal bias, a strict intention-to-treat technique will be considered in our analysis. Furthermore, the variable imputation method will be used to handle the missing data. After each intervention, an interim analysis will be performed by the main investigator.

### 3.11. Adverse Event Reporting

We will consider that a participant may likely identify a medication-related issue during pharmacist counseling, either in the intervention or control group. However, given the nature of the study and the data collection procedure, the chance of this occurring is unlikely. If any concerns do arise, the patient’s doctor will be promptly notified.

### 3.12. Pilot Study

To test the feasibility and acceptability of the protocol, a pilot study will be conducted in two community pharmacies, involving five patients from Bangladesh and Pakistan. Three investigators will provide feedback on the process based on the pilot study findings and experiences. We will then confirm and review our logistics and make any necessary changes to the intervention process.

### 3.13. Statistical Analysis

All outcomes will be analyzed using the intention-to-treat principle. Descriptive statistics will be used to present baseline characteristics and outcome variables. Percentages and frequencies will be used for categorical variables, and means and standard deviations will be used for continuous variables. Baseline characteristics between the two arms will be compared with chi-square or Fisher’s exact test, as appropriate. A multilevel mixed model will be used to compare the differential changes in each outcome across the time points (T1, T3, and T5) to measure the primary and secondary outcomes. We will analyze group changes (with individual pharmacies treated as a random effect) using multivariable mixed linear and non-linear regression for normally and non-normally distributed data to account for the cluster randomization effects. Point estimates from cluster-adjusted models will be reported with 95% confidence intervals. Additionally, subgroup analysis will stratify data based on hypertension-related complications and the number of associated medications to estimate the effect of the interventions. For all two-tailed statistical tests, *p*-value < 0.05 will be considered statistically significant. We will perform all statistical analyses using R (version 4.1) and Stata software (version 17.0).

A cost-effectiveness analysis will be performed on the average total cost incurred in both arms, and incremental cost-effectiveness ratios expressed as incremental cost per (1) 1, 5, or 10 unit (mmHg) decrease in blood pressure; (2) one hospital admission reduced; (3) 1 day of hospital stay reduced over the previous 6 months among the survivors; and (4) HRQoL using data from EQ-5D-5L and mortality. We will calculate all cost data in US dollars, using the non-subsidized cost as of the study’s commencement.

### 3.14. Ethical Approval and Dissemination

After obtaining ethical approval from Hitotsubashi University and informed consent from the participants, an equal number of participants will be recruited for the intervention and control groups. The participants will be informed about the strictly voluntary nature of their participation and the right to completely withdraw from the survey at any point, without needing to provide a reason or fearing any impact on their rights. They can refrain from answering any question if they feel uneasy. Participants will also be assured that their personal data will remain confidential at all stages, and their identification will not be disclosed. The study findings will be disseminated through local, national, and international conferences; multiple peer-reviewed journals; and reports to key stakeholders. Trial registration number: ClinicalTrials.gov NCT06148142. Registered on 27 November 2023. https://classic.clinicaltrials.gov/ct2/show/NCT06148142.

## 4. Discussion

This study protocol is developed as a quantitative approach to illustrate the feasibility of implementing a pharmacy-based health promotion program in South Asian community settings. The study aims to assess the impact and cost-effectiveness of education and counseling by CPs for controlling BP and promoting healthy lifestyle behavior among hypertensive individuals.

The evidence generated from this study is expected to aid in affirming the role of CPs as an integral part of primary care services to maximize the quality of provided care and match the specific needs of people in Bangladesh and Pakistan. CPs will be more confident in recognizing inadequate hypertension knowledge and medication adherence, and at-risk patients in the future. This will not only reinforce their role in improving the healthcare system but also lead to superior hypertensive patient outcomes.

This study will also exert significant impacts on hypertensive individuals, the community, and the healthcare system through the improved knowledge, healthier lifestyle choices, and capacity building of the CP to fill the gap in primary healthcare services. It is envisioned that pharmacies would become a central one-stop point in the community to retrieve hypertension-related information and management and obtain support among patients and caregivers. Apart from health and well-being, this study is anticipated to strengthen the patient–provider relationship between CPs and the community, which will be beneficial for tackling other chronic and communicable diseases. Lastly, with the decline in the rates of hypertension-related complications, non-adherence to medication, and hospitalization, the burden of the consequent cost of care will also be reduced. The study findings will thereby provide evidence on its sustainability and monetary value and hence inform stakeholders’ decisions in integrating this cost-effective model into the current community healthcare services.

## 5. Limitations

The main limitation could be the high dropout rate as patients may be too busy to attend an educational session, some might lose interest and discontinue during the study period, and some participants might miss their routine follow-up visits due to political unrest or any other unavoidable circumstance. Although the patients’ presence can never be guaranteed, the research team and the CP will try their best to contact and persuade patients to participate. Secondly, missing data can be an issue in this trial; however, the planned analyses will follow intent-to-treat methods to minimize bias from dropout over time. A repeated-measurement bias can also be expected as the same questionnaire will be used at both baseline and endline surveys. Finally, due to the short follow-up duration, the long-term benefits of this CP model may not be observed at length. Despite these limitations, the study findings can give a basis for improving the future care of hypertensive patients in primary healthcare facilities in South Asia.

## 6. Conclusions

Considering the positive outcomes of this study and the increasing number of pharmacists in South Asia, health policymakers and professional organizations in the near future can enact policies or regulations for CPs to offer these services effectively and continually. This will also provide evidence to further develop various CP-led innovative health services to streamline the referral systems more efficiently. Thus, strengthening the pillars of the community (i.e., primary health workforce) and developing such robust research evidence will not only ensure effective control of different health conditions but also reduce the burden of health expenditure in resource-poor settings.

## Figures and Tables

**Table 1:** Content of pharmacist-led counseling intervention.

	Contents	Description	Practical Application
1.	**Overview of hypertension**	The pharmacist will describe the basics of hypertension, the cause of hypertension, the risk factors of hypertension, and common symptoms of hypertension including the current global statistics on it.	Utilizing visual aids and statistics, pharmacists will provide a global perspective on hypertension to enhance patient understanding.
2.	**Complications of hypertension**	Pharmacists will describe the common health-related complications of hypertension.	By illustrating potential health outcomes, pharmacists will emphasize the importance of hypertension management.
3.	**Impact of hypertension on family’s healthcare expenditure**	The pharmacist will inform the participants how uncontrolled hypertension may increase the disease burden as well as contribute to increasing the total family’s healthcare expenditure.	Through examples and case studies, pharmacists will show the economic impact on families to encourage adherence to treatment plans.
4.	**Management of hypertension**	The pharmacist will inform the patients about the general and clinical management of hypertension.	Pharmacists will use guidelines and protocols to educate patients on effective hypertension management practices.
5.	**Importance of lifestyle modification**	The pharmacist will discuss lifestyle modifications in hypertension management with the participants, which will include cessation of smoking, reducing alcohol intake, maintaining body weight, reducing and managing mental stress, and performing physical activity regularly.	Interactive sessions and personalized plans will be used to help patients adopt healthier lifestyles.
6.	**Importance of medication adherence**	In this part, the pharmacist will advise the participants regarding the consequences of poor adherence to antihypertensive medication and how it reduces the ultimate clinical outcome.	Pharmacists will provide strategies to improve medication adherence, such as setting reminders and creating a medication schedule.
7.	**Importance of food habit on hypertension control**	The pharmacist will inform the patients how food habits impact hypertension control, which will include avoiding extra salt consumption in meals, avoiding saturated fat and trans fats, much intake of food and vegetables, etc.	Diet plans and nutritional counseling will be provided to help patients make healthier food choices.
8.	**Importance of regular blood pressure measurement on hypertension control**	The pharmacist will suggest to the participants the significance of timely blood pressure monitoring and how it helps a person become more aware of their body. Also, they will suggest how blood pressure measuring assists a doctor in making a quick diagnosis of a health issue.	Demonstrations on how to properly measure BP and track readings will be conducted to empower patients to monitor their health.

**Table 2:** Outcome measures and data collection time points.

	Screening, Enrollment and Randomization					Baseline	Follow-Up					Closed
	Wk 1	Wk 2	Wk 3	Wk 4	Wk 5	Month 0 (T1)	Month 3 (T2)		Month 6 (T3)	Month 9 (T4)		Month 12 (T5)
***Assessment and intervention***												
Selection criteria	x	x	x	x								
Informed consent	x	x	x	x								
Randomization					x							
Allocation					x							
***Physical examination (Control and intervention group)***												
Blood pressure measurement						x			x			x
BMI						x			x			x
Blood glucose measurement						x			x			x
***Baseline characteristics (Control and intervention group)***												
Sociodemographic profile						x			x			x
Dietary habits						x			x			x
Physical exercise habits						x			x			x
Knowledge of hypertension						x			x			x
Direct treatment cost information						x			x			x
Indirect treatment cost information						x			x			x
HRQoL (EuroQoL-5D)						x			x			x
***Intervention criteria (Only intervention group)***												
Audio–visual presentation						x			x			
Booklet distribution						x						
Phone call intervention							x			x		
***Outcome measurements (Control and intervention group)***												
*Primary outcome*												
Change in SBP and DBP									x			x
*Secondary outcome*												
BP controlled to target									x			x
Composite outcome of death or hospital admission									x			x
Improvement in knowledge									x			x
Changes in lifestyle									x			x
Changes in drug adherence									x			x
HRQoL gained									x			x
Cost per unit of BP reduction									x			x
Cost per HRQoL gained									x			x

HRQoL, health-related quality of life; SBP, systolic blood pressure; DBP, diastolic blood pressure; BP, blood pressure.

## Data Availability

The data follow the Guidelines for Accurate and Transparent Health Estimates Reporting. Requests for data-sharing will be considered by the corresponding author.

## Supplementary Materials

The following supporting information can be downloaded at: https://www.mdpi.com/article/10.3390/healthcare12141402/s1, Supplementary Files S1 and S2.
