# Supplementary material for: Effectiveness of a Community Pharmacy-Based Health Promotion Program on Hypertension in Bangladesh and Pakistan: Study Protocol for a Cluster-Randomized Controlled Trial
Source: Healthcare (Basel). 2024 Jul 15;12(14):1402. doi: 10.3390/healthcare12141402 (PMC11276715; doi:10.3390/healthcare12141402)
Supplement: Supplementary file 1 [file healthcare-12-01402-s001.zip › healthcare-3083715-File S1-Appendix1_Booklet.pdf]

eMethod 1

Pharmacy-based hypertension control program

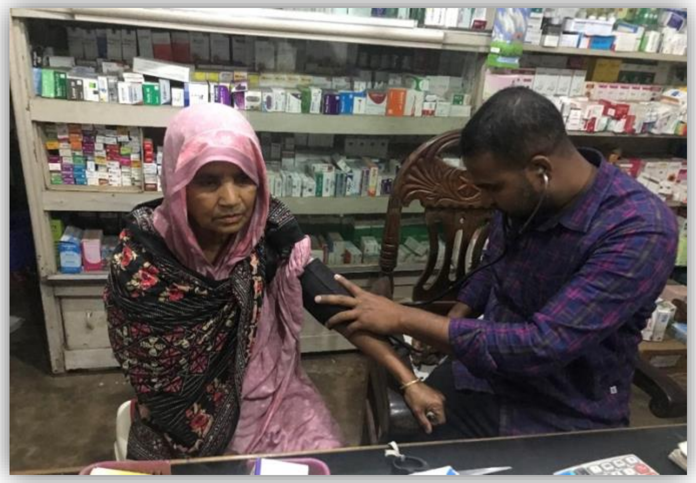

Name:
Village: **Union:** **District:**
Age (current age):
Gender:
Year of your hypertension started: ……..

	Blood pressure (mmHG)		Blood glucose (gm/dL)
Date [D/M/Y]	Systolic	Diastolic	

Global Public Health Research Foundation (Bangladesh)

&

Hitotsubashi University (Japan)

2023

[Type here]

What is hypertension?

Blood pressure (BP) is the force of circulating blood against walls of the body's arteries. Normal blood pressure is **120/80** mm Hg or lower. Hypertension is diagnosed when the force of blood exceeds the normal rate and BP readings are **140/90** or above on two different days.

According to World Health Organization (WHO), **1.13** billion people have hypertension around the globe and each year about **20** million people die worldwide due to hypertension-related complications.

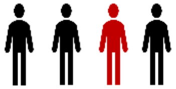

1in **4** men have high blood pressure

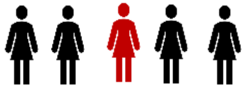

1in **5** women have high blood pressure

Sign and symptoms of hypertension

The chance of having high hypertension increases with age.

The common risk factors are-

- ✓ Excess dietary salt consumption
- ✓ Lack of physical activity
- ✓ Being overweight or obese
- ✓ Smoking
- ✓ Excess alcohol intake
- ✓ Low intake of fruits and vegetables
- ✓ Excess fatty food consumption
- ✓ Familial history of hypertension
- ✓ Excess mental stress and sleep disorder

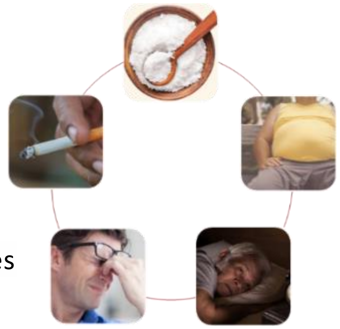

[Type here]

Causes of Hypertension

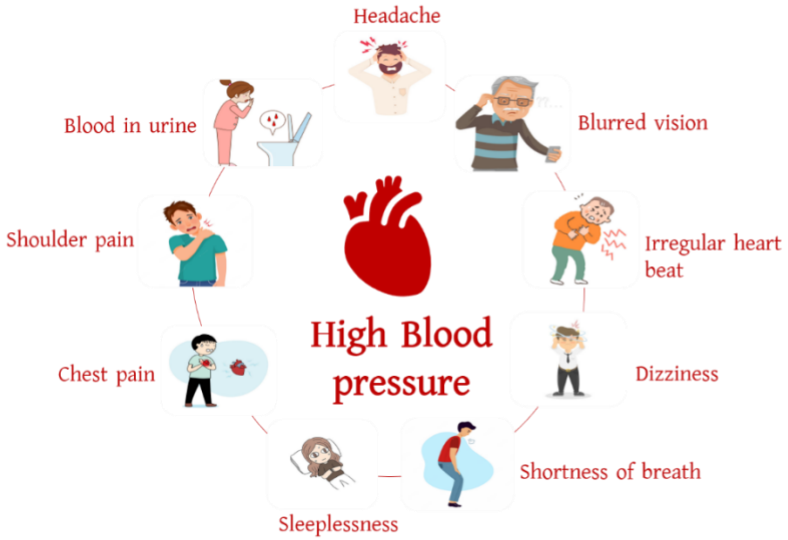

Complications of hypertension

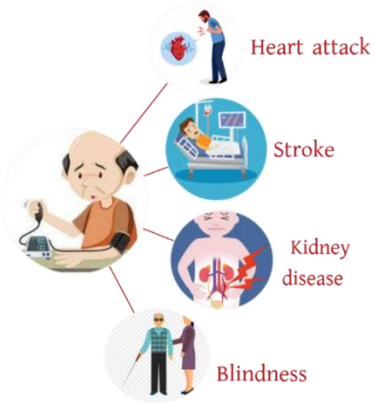

Hypertension increases family’s healthcare expenditure

The high cost of care (drugs, treatment, complications, hospitalization) ultimately affects the family's monthly expenditures and lowers the quality of life.

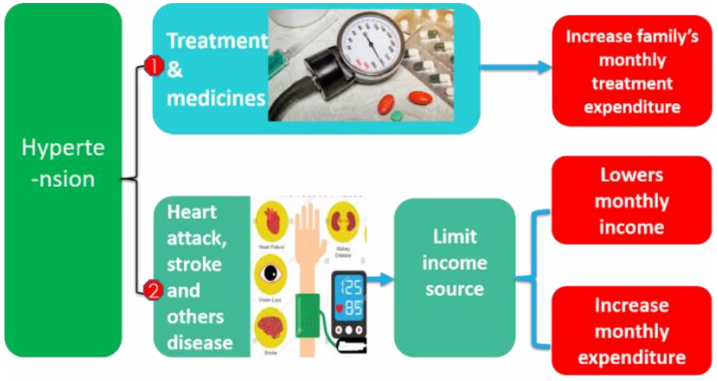

Steps of Lifestyle modifications

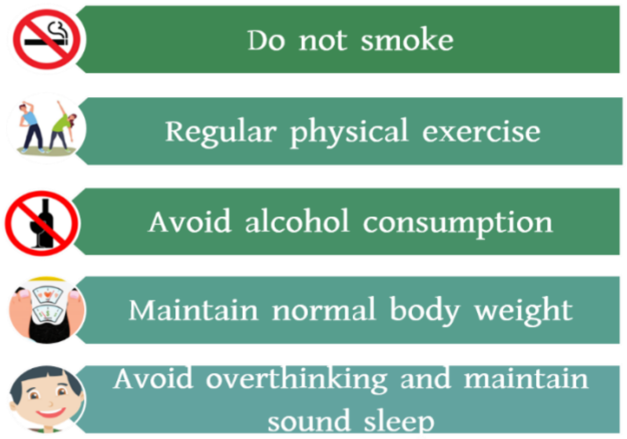

Management of hypertension

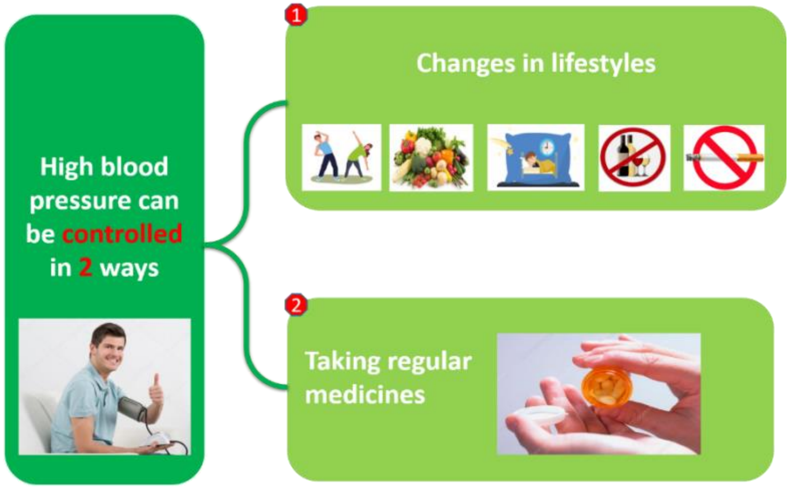

Benefit of taking regular anti-hypertensive medicine

Skipping doses or taking them infrequently increases the risk of hypertension complications by **50% to 80%.**

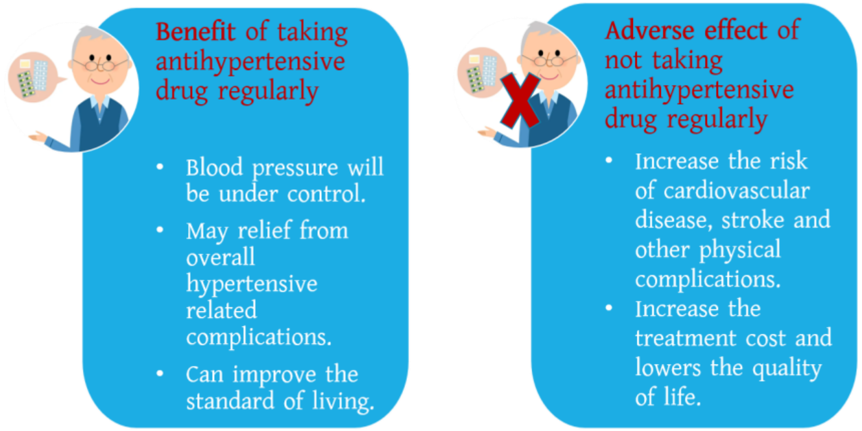

[Type here]

Changes in food habit to control blood pressure

Eat This	Limit This
Vegetables	Fatty meats
Fruits	
Whole grains	Full-fat dairy
Fat-free or low-fat dairy	
Fish	Sugar sweetened beverages
Poultry	
Beans	Sweets
Nuts & seeds	
Vegetable oils	Sodium intake

Benefit of regular measurement of blood pressure

By measuring blood pressure regularly:

- ✓ One can understand whether lifestyle changes and medications are working effectively to control the blood pressure.
- ✓ It will also help in diagnosing cardiovascular and other diseases.

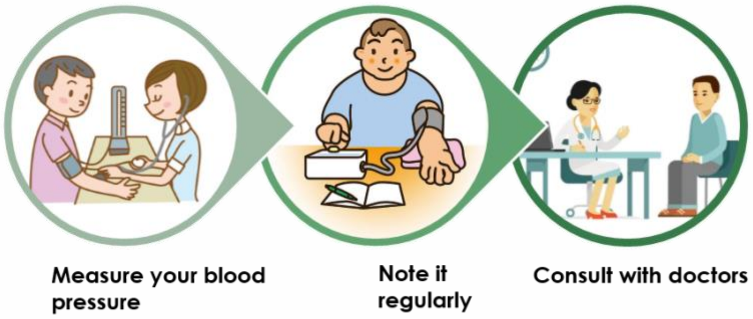

Measure blood pressure by yourself or by others and **note it**.

If you observe any **abnormalities** in your blood pressure, **please visit a doctor** at the earliest.

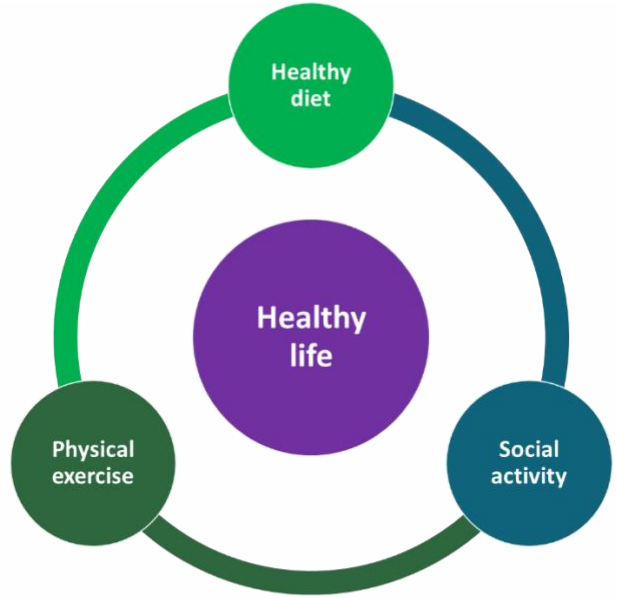

Live a disciplined life, keep blood pressure under control

[Type here]

Global Public Health Research Foundation (GPHRF)

Global Public Health Research Foundation (GPHRF) is a non-profit organization whose mission is to improve population health at global and national level through delivering high quality research, evidence and promoting healthy lifestyle.

Mission: GPHRF aims to improve population health and develop leaders in public health through research, education, training and collaborating with national and global health agencies.

Activities

- To undertake research project on global and public health issues sponsored by public and private organization independently or in collaboration with national or international organization.
- To publish high quality global and public health research, both of fundamental and applied interest in international and national journal.
- To organize symposiums, seminars and conferences in the fields of health science to promote global and public health research and to disseminate knowledge with different stakeholders.
- To provide short term courses and training programs on the fields of public health, epidemiology, systematic review and meta-analysis, and health financing research to interested and qualified persons from outside the GPHRF.
- To provide consultation services and policy advices on public health to the governmental and non-governmental organizations and all other organizations which seek such assistance.

GPHRF

Global Public Health Research Foundation (GPHRF)
House #30, Road #12, Sector #10, Uttara, Dhaka-1230 Bangladesh.
Phone: +8802 55092268
Mobile: +8801797 438880, +8801797 438889
E-mail: info@gphrf.org
Website: www.gphrf.org

Hitotsubashi University

Hitotsubashi University situated in Tokyo of Japan, was established as a center of education and research embracing every field of the social sciences, which make up the learning of civil society.

Mission: To establish a solid foundation for research and for the creation and study of new fields in the social sciences, and to develop, accumulate, and widely publish intellectual and cultural property rich in originality.

Activities:

- To provide education and conduct research in a spirit of autonomy and intellectual integrity cognizant of the University's social responsibility and duly respecting law and ethics.
- To provide education and conduct research in a spirit of autonomy and intellectual integrity cognizant of the University's social responsibility and duly respecting law and ethics.
- To provide education and conduct research in a spirit of autonomy and intellectual integrity cognizant of the University's social responsibility and duly respecting law and ethics.
- To provide education and conduct research in a spirit of autonomy and intellectual integrity cognizant of the University's social responsibility and duly respecting law and ethics.

HITOTSUBASHI UNIVERSITY

Hitotsubashi University

2-1 Naka, Kunitachi, Tokyo 186-8601, Japan

Phone: +81-42-580-8000, wwwadm@ad.hit-u-ac.jp

Website: https://www.hit-u.ac.jp

[Type here]
