# Supplementary material for: Effectiveness of a Community Pharmacy-Based Health Promotion Program on Hypertension in Bangladesh and Pakistan: Study Protocol for a Cluster-Randomized Controlled Trial
Source: Healthcare (Basel). 2024 Jul 15;12(14):1402. doi: 10.3390/healthcare12141402 (PMC11276715; doi:10.3390/healthcare12141402)
Supplement: Supplementary file 1 [file healthcare-12-01402-s001.zip › healthcare-3083715-File S2-Appendix_2_Community pharmacist training manual.pdf]

Pharmacy based Hypertension control program

Courtesy of:

Purpose:

The main purpose of this project is to measure the impact of pharmacy-based counselling for hypertension control and it's cost-effectiveness in Bangladesh, India, and Pakistan.

Benefits:

- Improve the knowledge for blood pressure control and lifestyle
- Improve the efficiency of community pharmacist in patient management
- Building strong patient-provider relationships within the community
- Reduce hypertension-related complications, medication nonadherence, frequency of physician visits, and hospital admissions
- Reduction of the overall burden of health expenditure

GPHRF GLOBAL PUBLIC HEALTH RESEARCH FOUNDATION

What is hypertension?

In general, for a healthy person normal blood pressure is 120/80 mmHg. If someone’s blood pressure is more than this limit that condition defined as hypertension.

That means, whenever someone's blood pressure is more than **140/90** mmHg then he/she identified as hypertension patient. Sometimes blood pressure also called as ‘pressure’.

Although blood pressure **may vary** according to age.

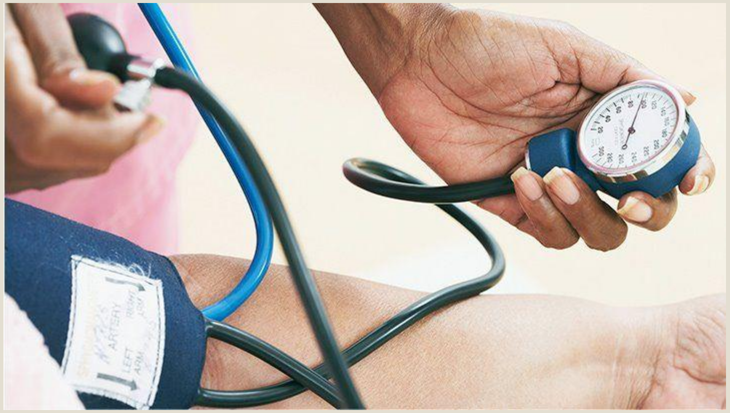

GPHRF GLOBAL PUBLIC HEALTH RESEARCH FOUNDATION

All over the world, about **1.3** billion suffer from hypertension and about **20** million people worldwide die from hypertension-related complications.

1 in 5 women have hypertension

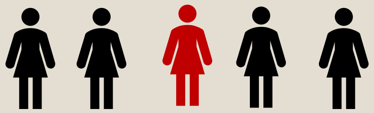

1 in 4 men have hypertension

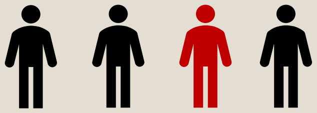

Source: World Health Organization (2022)

https://www.who.int/health-topics/hypertension#tab=tab_1

GPHRF GLOBAL PUBLIC HEALTH RESEARCH FOUNDATION

Sign and symptoms of hypertension?

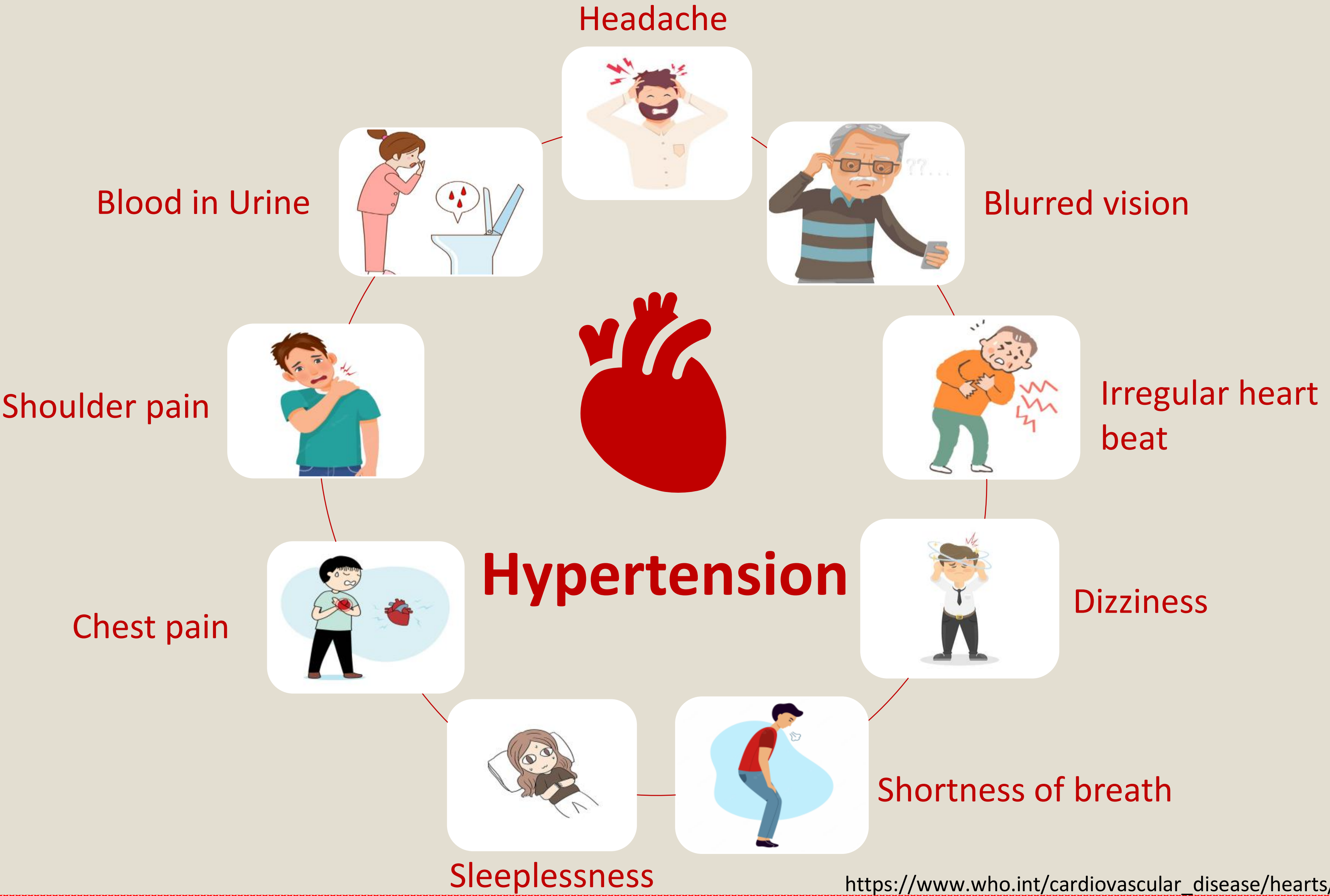

https://www.who.int/cardiovascular_disease/hearts/en

GPHRF GLOBAL PUBLIC HEALTH RESEARCH FOUNDATION

Causes of Hypertension?

The chance of having high hypertension increases with age.

The common risk factors are-

- Excess dietary salt consumption
- Lack of physical activity
- Being overweight or obese
- Smoking
- Excess alcohol intake
- Low intake of fruits and vegetables
- Excess fatty food consumption
- Familial history of hypertension
- Excess mental stress and sleep disorder

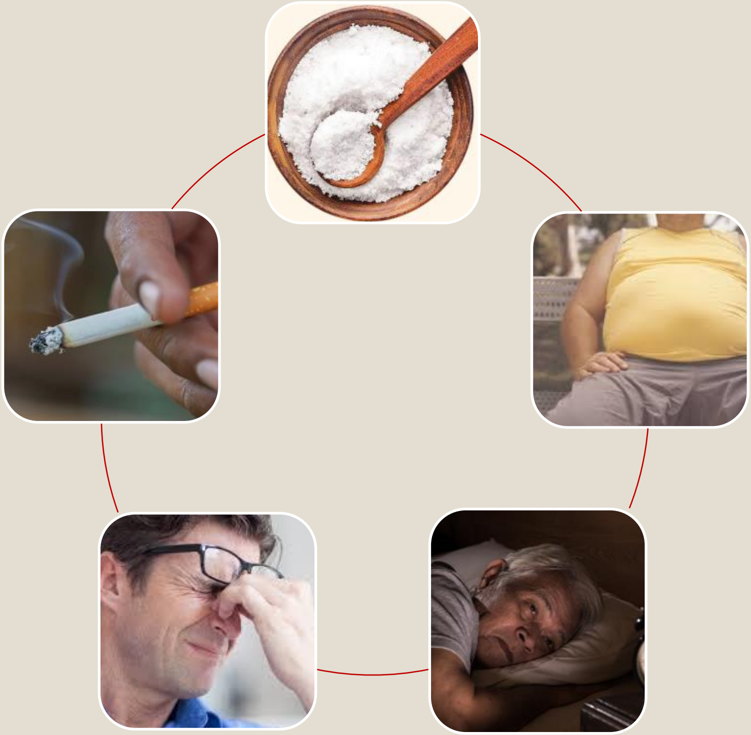

https://www.who.int/cardiovascular_disease/hearts/en

GPHRF GLOBAL PUBLIC HEALTH RESEARCH FOUNDATION

Complications of hypertensions?

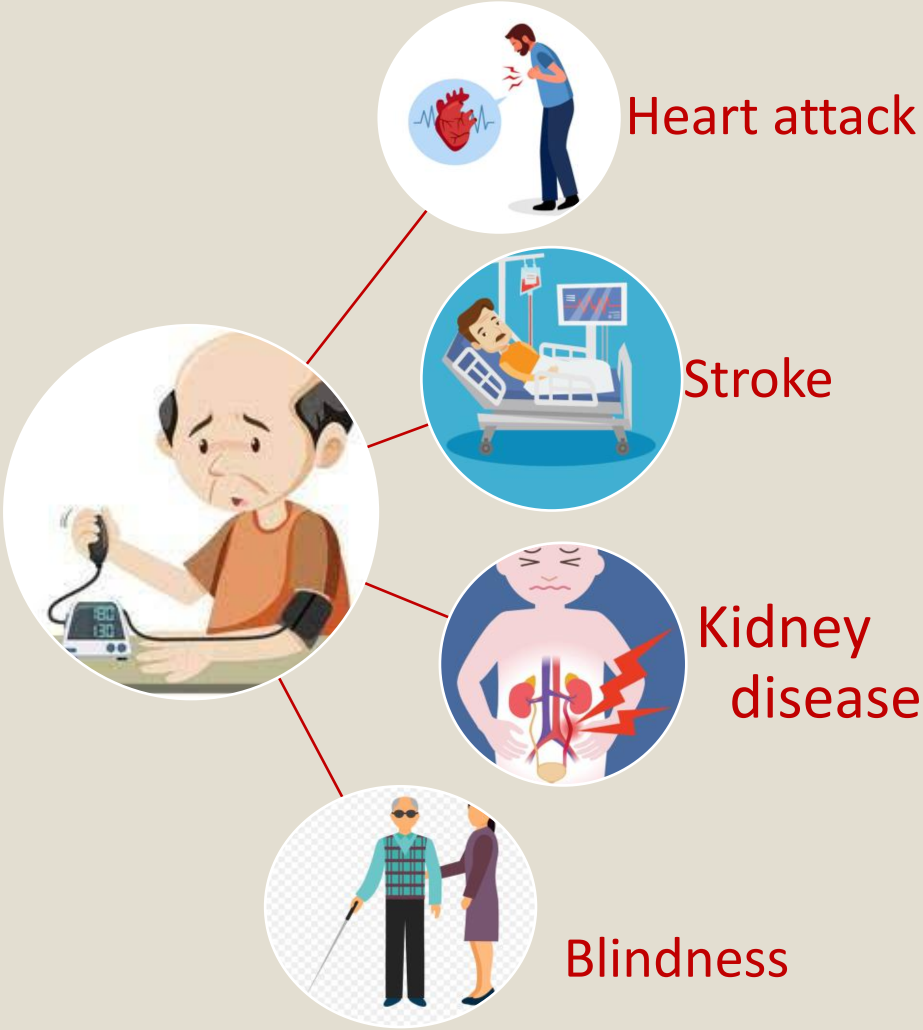

https://www.who.int/cardiovascular_disease/hearts/en

GPHRF GLOBAL PUBLIC HEALTH RESEARCH FOUNDATION

Hypertension increases family's healthcare expenditure

Hyperte-nsion

1 Treatment & medicines

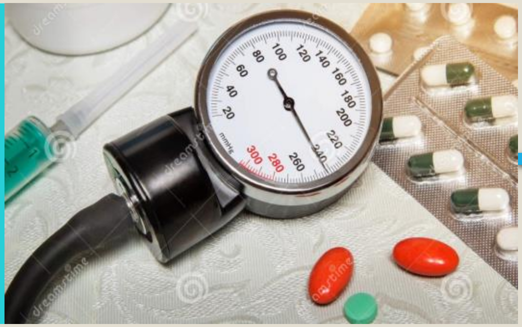

Increase family's monthly treatment expenditure

2 Heart attack, stroke and others disease

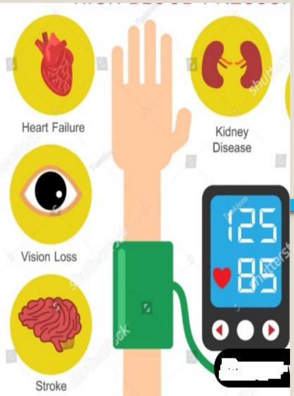

Limit income source

Lowers monthly income

Increase monthly expenditure

Management of high blood pressure

High blood pressure can be controlled in 2 ways

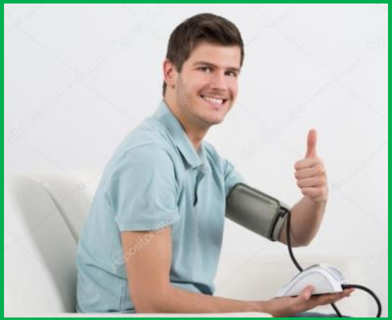

1

Changes in lifestyles

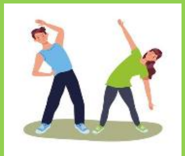
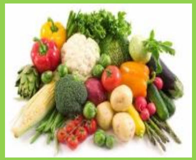
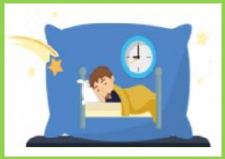
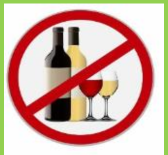
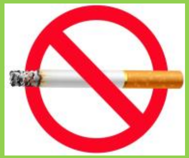

2

Taking regular medicines

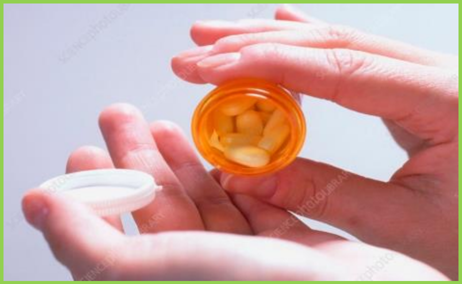

Important of lifestyle modifications in hypertension control

Changes in lifestyle modifications lower blood pressure and hypertension related complications:

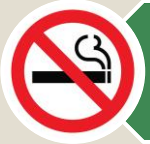

- Do not smoke

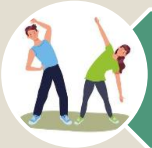

- Do regular physical exercise

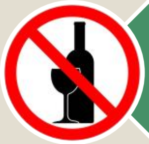

- Avoid alcohol consumption

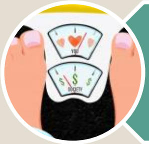

- Maintain normal body weight

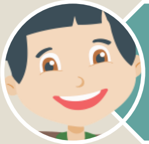

- Avoid over thinking and maintain sound sleep

GPHRF GLOBAL PUBLIC HEALTH RESEARCH FOUNDATION

Important of taking anti-hypertension medicine regularly

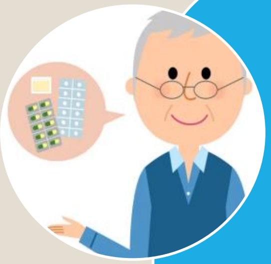

Benefits of taking antihypertensive drug regularly

- Blood pressure will be under control.
- May relief from overall hypertensive related complications.
- Can improve the standard of living.

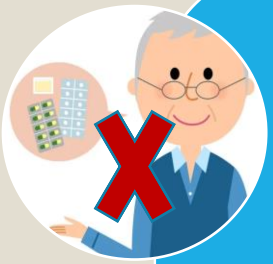

Adverse effect of not taking antihypertensive drug regularly

- Increase the risk of cardiovascular disease, stroke and other physical complications.
- Increase the treatment cost and lowers the quality of life.

Important of changing food habit in hypertension control

GLOBAL PUBLIC HEALTH RESEARCH FOUNDATION

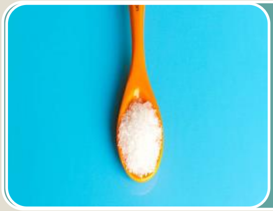

Limit the salt intake.

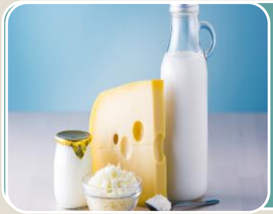

Avoid butter and cheese

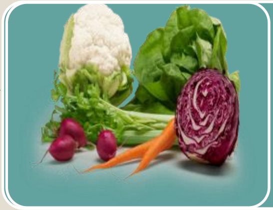

Eat more vegetables and fruits

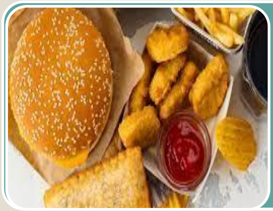

Avoid high fat diet

Importance of Regular measurement of blood pressure

GPHRF GLOBAL PUBLIC HEALTH RESEARCH FOUNDATION

By measuring blood pressure regularly:

- One can understand whether lifestyle changes and medications are working effectively to control the blood pressure.

- It will also help in diagnosing cardiovascular and other diseases.

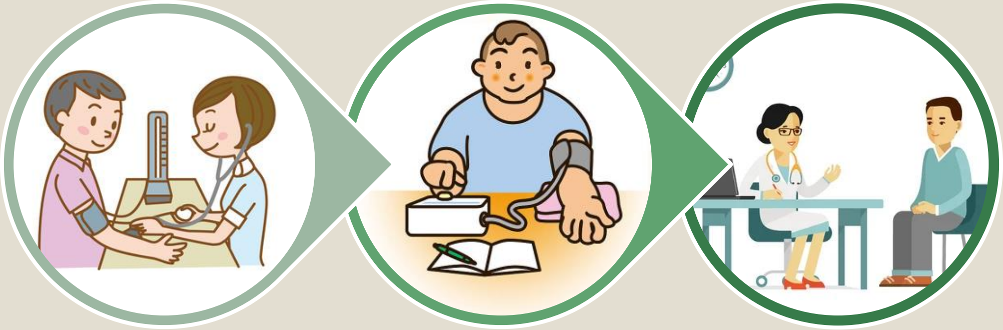

Measure blood pressure regularly

Note it

If observe any abnormalities consult with doctor

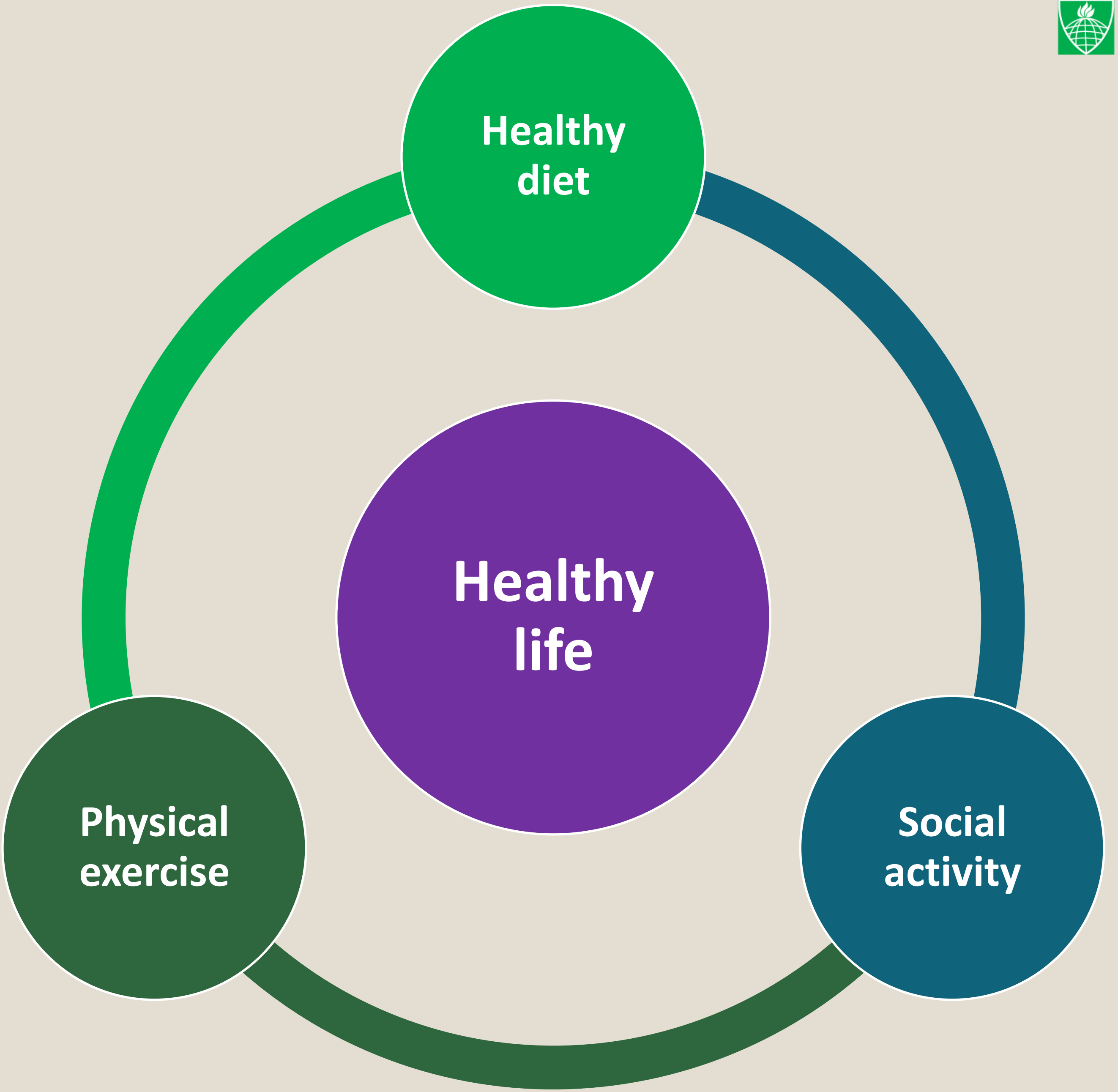
Healthy diet
Healthy life
Physical exercise
Social activity

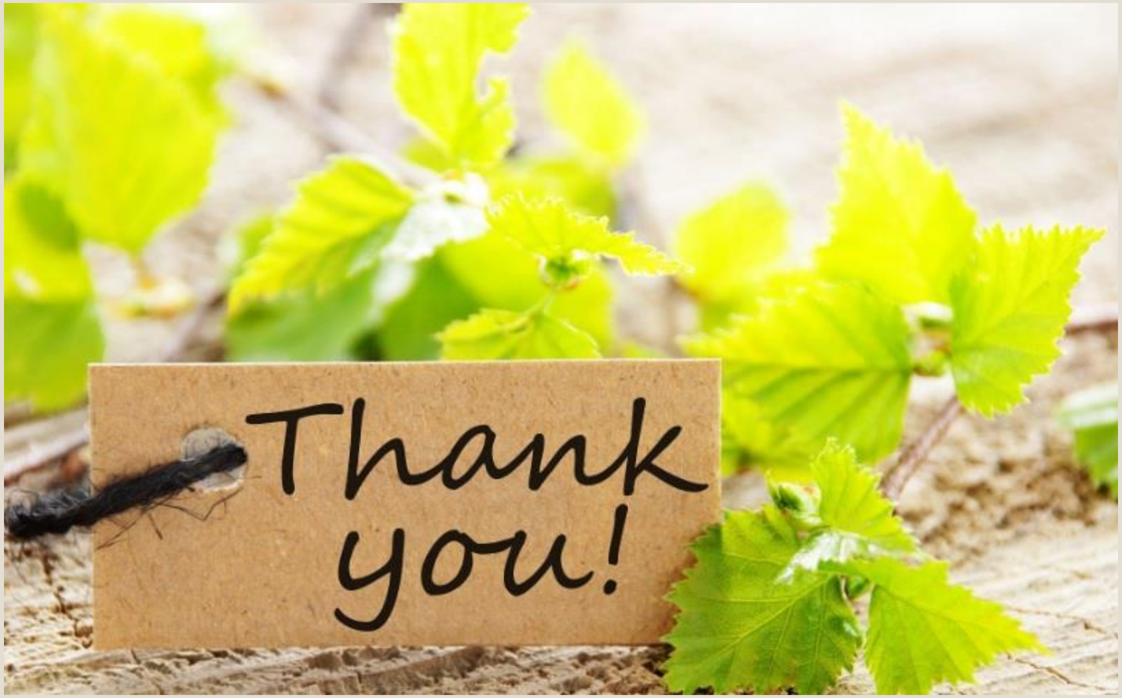
Thank you!
